# Nobody says to you “come back in six months and we’ll see how you’re doing”: a qualitative interview study exploring young adults’ experiences of sport-related knee injury

**DOI:** 10.1186/s12891-020-03428-6

**Published:** 2020-07-01

**Authors:** Ross Watkins, Georgina Young, Max Western, Keith Stokes, Carly McKay

**Affiliations:** 1 University of Bath, Claverton Down, Bath, BA2 7AY UK; 2 Centre for Sport, Exercise and Osteoarthritis Research Versus Arthritis, Bath, UK

**Keywords:** Young adults, Knee injury, Self-management of health, Digital health

## Abstract

**Background:**

Regular exercise is vital for overall health, and key to the maintenance of joint health. However, whilst people are encouraged to participate in sport and exercise, many are unaware that they could be at risk of developing post-traumatic osteoarthritis (PTOA) in the years following sport-related injury. Younger adults (< 40 years) with PTOA can experience declining quality of life, comorbid health conditions, and symptoms that place a chronic burden on health services. Conserving knee health through careful self-management in the latency period between injury and the onset of PTOA may help to delay disease progression. In this regard, the development of self-management interventions can be facilitated by understanding the post-injury experiences of young adults and their attitudes towards joint health.

**Methods:**

Semi-structured interviews were conducted with 13 young adults following a sport-related knee injury to explore their experiences of injury, and their attitudes and perceptions of self-managing knee health. The interviews were audio-recorded, transcribed and analysed systematically using an inductive approach.

**Results:**

Four themes pertaining to participants’ experiences were identified: [1] perceptions of current care provision; [2] long-term impact of knee injury; [3] motivation to conserve knee health; and [4] opportunities for supplementary support. The expression *“Nobody says to you ‘come back in six months and we’ll see how you’re doing’”* personifies the long-term impact of knee injury on young adults and a paucity of care provision.

**Conclusion:**

Participants did not perceive that they had adequate care in the aftermath of knee injury, leading to a sense of frustration and uncertainty. This had implications for continued participation in sport and exercise, negatively impacting their athletic identity and sense of wellbeing. Activity tracking, symptom monitoring, advice provision and peer support were identified as tools to enable individuals to self-manage knee health.

## Background

The physical and psychological benefits of sport and physical activity (PA) in young people (and through the life course) are well-recognised [1]. However, participation in sport and recreational activities also brings with it the risk of injury. Sports injury often has a short-term adverse effect on health-related quality of life, including pain and reduced physical function, as well as an impact on emotional wellbeing [2]. Longer-term, the negative effects of injury may result in the cessation of sport participation or PA due to ongoing symptomology or the fear of injury recurrence [3]. For example, only about half of sports participants return to competitive sport following anterior cruciate ligament (ACL) rupture [4]. Moreover, a sports injury in young adulthood may lead to the development of post-traumatic osteoarthritis (PTOA) – a type of osteoarthritis (OA) affecting young people with a history of joint injury including articular fracture, ligamentous rupture, joint dislocations, or meniscus tear [5]. The knee is the joint most commonly affected by OA, accounting for 80% of the global OA burden [6] and affecting 20% of adults over the age of 45 in England [7]. Meta-analyses have reported a three- to seven-fold increased risk of PTOA in individuals ten to 20 years post-knee injury [8]. Given the latency period between knee injury and the onset of PTOA, there may be an opportunity to delay or prevent it through careful management of factors that have a deleterious effect on joint health. In young adults with existent knee-related symptoms (e.g., pain, swelling, stiffness), careful management of knee health may help to ameliorate poor knee function and associated quality of life [9].

There is growing evidence that in the 3 to 10 years after a sport-related knee injury, young adults are more likely to become overweight or obese, less physically active, and develop magnetic resonance imaging (MRI)-defined OA compared with their uninjured peers [10, 11]. Such findings suggest that young adults with a history of knee injury may compound their risk of PTOA through exposure to other modifiable risk factors that may accelerate the rate of progression to OA (e.g., obesity, inactivity, and altered joint loading) [10]. Given the role of these modifiable risk factors in disease progression, a greater understanding of young adults’ attitudes and beliefs about their knee health after injury is paramount. Supportive rehabilitation which meets individuals’ physical, psychosocial, and informational needs will establish a positive trajectory for longer-term health and address these risk factors directly. This includes addressing the negative effects of injury which may result in sport or physical activity cessation due to the fear of injury recurrence [3] or the persistence of knee difficulties which leave individuals unable to adapt to an active lifestyle post-injury [12].

Where OA is established, guidance from experts in the field emphasises the need for management using a combination of behavioural and clinical strategies [13]. Core non-pharmacological therapies include self-management education, land- or water-based aerobic exercise, muscle strength training, neuromuscular exercise, lifestyle PA, and weight management, as well as other evidence-based techniques such as increasing joint range of motion with manual therapy and biomechanical interventions such as braces [14]. Unfortunately, there are major gaps in both OA prevention and the use of evidence-informed therapies in the UK and internationally [15, 16]. For example, studies indicate that healthcare providers often do not include recommendations for exercise and weight management as part of OA management [15]. Therefore, there is a clear need to develop and implement interventions that promote evidence-informed OA prevention and management. Implicit within such interventions is the notion that individuals identified as at risk of PTOA, or with established PTOA, should be provided with the tools to self-manage their joint health, thereby reducing the burgeoning reliance on frontline health services.

In order to inform the development of interventions that enable individuals to self-manage knee health, it is important to understand target users’ experiences post-injury, explore their attitudes towards joint health, and identify opportunities for increased self-management. These data will help to develop an intervention, as well as help inform a protocol that is acceptable to users – including features that target user behaviour and offer options for personalised self-management strategies. This is akin to the Person-Based Approach, which uses qualitative research with target users to formulate guiding principles, which in turn specify the design objectives and key components of an intervention [17]. By systematically investigating the attitudes and perceptions of target users, this approach helps to ensure that interventions are acceptable, feasible, meaningful and optimally engaging [17, 18]. Therefore, the current study aimed to:

1. Understand young adults’ experiences following knee injury;
2. Elicit attitudes and perceptions of knee health; and
3. Explore how self-care could augment current care provision.

## Methods

Ethical approval for the study was given by the Research Ethics Approval Committee for Health at the University of Bath (Reference Number: EP 18/19023). Written consent was obtained from all participants prior to interviews.

### Participants

Male and female amateur sports participants aged 18 to 35 were invited to participate in this study. Participants were recruited through existing networks including University sports teams and societies, as well as community groups and personal networks. Individuals were eligible to participate if they identified as non-professional sports participants and had suffered a sport-related knee injury within 1 to 10 years or an anterior cruciate ligament (ACL) rupture within the last 18 months to 10 years (to account for typical injury recovery times). An email was sent to potential participants with information about the study, advising them to contact the research team if they wished to participate. Recruitment ceased once theoretical saturation was reached (i.e. when no new themes emerged from the data). A maximum variation sample was recruited using a combination of purposive, convenience, and snowballing techniques [19]. It was anticipated that between 10 and 15 participants would give a sufficient range of experiences and depth of data to reach theoretical saturation [20, 21]. Each participant who gave their consent to take part in the study was assigned a unique reference number (e.g., YA001).

### Semi-structured interviews

One-to-one semi-structured interviews were conducted by the lead researcher (RW) with a focus on participants’ experiences of knee injury and self-management of their knee health. An interview topic guide (Additional file 1) was used to steer the interview dialogue. Participants were free to say as much as they wished. As important issues or themes emerged, they were included in subsequent interviews and structured further questioning in order to facilitate the development of a theory. As data was collected, repeated ideas (e.g. views and opinions) were tagged with codes, which could then be grouped into concepts and/or categories. It was an iterative, emergent process, which helped to conceptualise the experiences of young people following knee injury. The interviews were audio recorded and transcribed verbatim, facilitating analysis and the development of theory. Interviews lasted between 30 and 60 min.

### Data analysis

The data collated from the interviews were analysed using inductive Thematic Analysis [22, 23]. The aim of the analysis was to organise the data in a meaningful way so as to be able to develop a theory of the forms, functions and consequences of knee injury on PA, quality of life, and beliefs about self-management of knee health. The transcripts were first reviewed several times for familiarisation, before coding each meaning unit with a data-driven label. Codes were then explored for interconnections, and related codes grouped into primary clusters. In order to ensure that the qualitative research was rigorous, trustworthy and credible [24], two researchers (RW,GY) analysed the transcripts independently. The lead researcher (RW) then organised the coded data into themes and a second researcher reviewed them for coherence (GY). Differences were resolved through discussion of the themes and their interpretations. The provisional themes were then refined after discussion with all of the authors. NVivo (QSR International; Version 12 Pro) was used for data management and analysis.

## Results

Seven men and six women took part in interviews. Further recruitment was not undertaken as new themes in the data were not emerging. The age of participants ranged from 19 to 35 with a mean age of 27 (standard deviation 4.6). All participants reported acute knee injury as a result of playing local or national level amateur rugby, netball, football or roller derby within the last 1 to 10 years (18 months to 10 years in the case of ACL rupture). Five of the participants had suffered an ACL rupture followed by surgical reconstruction (ACL-R), three participants had injured their medial collateral ligament (MCL) with two requiring surgical intervention, two participants had dislocated their patella in addition to ACL rupture and posterior cruciate ligament (PCL) tear in respective cases, and one participant had ruptured their ACL and torn their MCL and PCL. The remaining two participants had not received a confirmed radiographic diagnosis but had experienced ongoing symptoms following acute knee injury (see Table 1).

**Table 1:** Participant characteristics

Participant	Age	Gender	Primary sport	Injury^a^	Time since injury	Time out of sport	Ongoing symptoms
YA001	24	Male	Rugby	ACL rupture with PCL and MCL tear followed by ACL-R	20 months	Changed sport to rowing 1 year post-injury	Slightly reduced range of motion
YA002	35	Male	Football	Undiagnosed knee injury (no surgery)	9 years	Changed sport to tennis 6 months post-injury	Occasional flare-ups
YA003	35	Female	Netball	ACL rupture followed by ACL-R	2 years	18 months	None
YA004	32	Female	Roller Derby	ACL rupture followed by ACL-R	6 years	10 months	Occasional flare-ups
YA005	28	Male	Football	MCL tear (no surgery)	2 years	6 months	None
YA006	25	Male	Rugby	MCL and meniscus tear followed by surgical repair	18 months	8 months	Some weakness, pain after heavy loading
YA007	31	Male	Football	Undiagnosed knee injury (no surgery)	3 years	2 years (reduced participation)	Occasional pain and weakness
YA008	26	Male	Rugby	MCL and meniscus tear followed by surgical repair	2 years	9 months	Some weakness, soreness post-game
YA009	24	Male	Rugby	Dislocated patella with PCL tear (no surgery)	6 years	4 months	None
YA010	19	Female	Netball	ACL rupture followed by ACL-R	2 years	15 months	None
YA011	23	Female	Rugby	Dislocated patella and ACL rupture followed by ACL-R	2 years	18 months	Occasional pain and weakness
YA012	25	Female	Netball	ACL rupture followed by ACL-R	18 months	Yet to return	Occasional pain and weakness
YA013	27	Female	Netball	ACL rupture followed by ACL-R	2 years	1 year	None

^a^ All injuries listed where confirmed with MRI scan

As well as describing various procedural aspects of treatment and care following injury, participants gave nuanced accounts of their experiences post-injury and the impact this had on daily life. Four overarching themes emerged from the analysis pertaining to these experiences: [1] perceptions of current care provision; [2] long-term impact of knee injury; [3] motivation to conserve knee health; and [4] opportunities for supplementary support. Each theme included sub-themes which are presented with illustrative data extracts (see Table 2). The first theme is *perceptions of current care provision* in which participants reflected on the experiences of the treatment they received from healthcare professionals following knee injury. Theme 2 is *long-term impact of knee injury*, capturing the ways in which participants’ attitudes and beliefs have shifted post-injury. Theme 3, *motivation to conserve knee health*, comprises participants’ explicit and implicit reasons from maintaining health and wellbeing, whilst the fourth theme, *opportunities for supplementary support*, encapsulates participants’ views on how current care provision could be augmented. Anonymised participant quotes are used to illustrate themes.

**Table 2:** List of themes and sub-themes

Themes	Sub-themes
Perceptions of current care provision	Everybody seems to have been treated differently Nobody says to you “come back in six months and we’ll see how you’re doing”
Long-term impact of knee injury	I am always going to be wary now It was a whole lifestyle overhaul for me
Motivation to conserve knee health	Do what you can, while you can I just like having that something that I can say is mine
Opportunities for supplementary support	It could be a diagnosis kind of thing I wasn’t the only one having problems

### Theme 1. Perceptions of current care provision

#### “Everybody seems to have been treated differently”

Participants had mixed experiences of treatment following knee injury. On the one hand, they reported a lack of consistency in treatment by Consultants: *“One of the things that I’ve noticed from the people that I’ve spoken to is that everybody seems to have been treated a bit differently”* (YA011) and this did not give reassurance that they were provided with the best treatment option. Additionally, participants described how Consultant subjectivity in the context of individual treatment plans led to confusion and a sense that participants had not been properly informed. Much of this confusion centred on decisions over the merits of surgical intervention, particularly post-ACL injury:

*“That is the only thing I would have changed, I think I must have seen five surgeons in the space of two months, each with a different opinion and I think I just felt like I was in the dark about what was happening.”* (YA009)

Mixed messages also extended to the diagnosis of knee injury, where frustration was coupled with a sense that a lack of clarity was delaying the process of recovery: *“I just want someone to tell me what’s wrong with it. It’s been three years now, so it’s a bit embarrassing cos it’s been so long, but I just want someone to say, ‘this is what’s wrong’”* (YA007)*.* Delays in treatment (and diagnosis) also had repercussions for participants’ work and family life with uncertainty over when time would need to be taken off, and for how long. Slow progress was also attributed to the National Health Service (NHS),^1^ which did not afford participants the speed and flexibility of private healthcare: *“I decided to go privately because of the delays of going through the NHS and also not having the flexibility with work etc.”* (YA004). Participants reported paying privately for diagnostic scans, private appointments with Consultants, and private physiotherapy in order to expedite the treatment process. Post-injury and/or post-operative rehabilitation provided by Physiotherapists was also deemed by some participants to be inadequate. As well as the delays associated with physiotherapy referral and the lack of appointment flexibility, participants bemoaned the standardised approach taken by NHS Physiotherapists who did not necessarily cater for younger adults planning to return to sport: *“I definitely felt that it was like, well, we know you go to the gym and stuff you just kind of crack on, on your own sort of thing.”* (YA003).

Participants did discuss the benefits of group rehabilitation classes offered by healthcare providers. These were described as knee clinics for people who had undergone knee surgery, predominantly ACL reconstruction. Although the classes were attended by a mixed demographic, participants described how they “*really quite liked that set up and being part of a group”* (YA004) and how it was “*nice to be surrounded by people going through the same thing”* (YA009).

#### “Nobody says to you “come back in six months and we’ll see how you’re doing”

Aside from the group classes available in some clinical settings, participants were typically offered up to six sessions of physiotherapy post-injury and/or post-surgery on the NHS. However, regardless of individual treatment pathways, participants described the definitive nature of care provision following knee injury, asserting that there is a lack of provision over the longer-term:

*“Once you have been discharged from physio no one says to you ‘come back in six months and we’ll see how you’re doing’. Once you’ve been discharged, you’ve been discharged.”* (YA006)

Some participants resorted to supplementing their physiotherapy privately, but this was not a feasible option for all, and there was a general consensus that informed self-management of knee health was required beyond the point of discharge. The limited (and infrequent) number of appointments available with Physiotherapists also compromised participants’ confidence in following prescribed exercise programmes. Some participants reported that they felt unable to carry out exercises correctly because they had not been instructed to do so properly, or that they were overwhelmed by the information they were given: “*I also didn’t feel like there was a lot of clarity in the programme that I was given, it’s quite a lot, its three sheets of exercises”* (YA003)*.* Some of this lack of clarity hinged on uncertainty over when and how to progress with exercise programmes as joint strength and functionality improved. More broadly, participants voiced a desire for exercise programmes that extended beyond rehabilitation: “*it would have been nice for the couple of years after just to have had one appointment every six months as like a follow-up”* (YA008) in order that they could “*maintain the strength in the knee” based on “specific and clear information”* (YA002).

Longer-term management of knee health also presented participants with some uncertainty about where to seek advice and support. “Little niggles” and stiffness around the knee were perceived as an annoyance but because it didn’t stop participants “*getting up in the morning*”, they appeared to accept the discomfort. Participants acknowledged that *“people don’t want to go to the doctor because they think they are wasting their time”* (YA006) and there was a reluctance amongst participants to visit the General Practitioner (GP) because they didn’t perceive their knee symptoms as being serious enough to warrant an appointment:

*“I could definitely go back to the GP and make a big deal of it, but I always think well there’s probably people that are in a worse situation than me and it probably won’t be worth doing that as I can walk and I can exercise, but I can’t really do it the way that I want, but at the moment it’s not a huge issue.”* (YA002)

### Theme 2. Long-term impact of knee injury

#### “I am always going to be wary now”

Participants described how the experience of knee injury had changed their outlook. They sensed a perennial risk of re-injury as a result of feedback from healthcare professionals: “*I am aware of it, in the back of my mind, because I was told by the surgeons at the time that it probably will happen again* (YA008), as well as personal experience of having witnessed peers with recurrent knee injury. This had affected their knee confidence and prompted behaviour change: *“It’s in my head …. ever since then I’ve been thinking that maybe I should strap it up (knee) just as a precautionary sort of measure.”* (YA006). Knee injury had also prompted them to reassess their participation in sport, the level at which they play, and the amount of risk they were prepared to take with their future knee health. Despite a reluctance to mediate their commitment to their sport, they appreciated that a cautionary approach may benefit them in the long-term:

*“With the experience I have had, I think I am going to be a lot more wary, more cautious, with a) what I am doing and b) what I may have to turn it down, or say it was going back to rugby training, I would have to be a lot more sensible. Obviously, it is not what I want to do but it is going to help me in the long run physically.”* (YA010)

Moreover, participants acknowledged the wider implications of re-injury or degenerative knee health, asserting that knee problems had the potential to impact work, income, and life at home with their children:

*“So not to hurt myself anymore because I’ve always got that in the back of my mind that if I hurt myself I will be back out of action for work and obviously, in terms of work it’s the income as well, and with the children and playing with them.”* (YA008)

For some, continuing to play sport was a risk worth taking, as long as that participation continued to be enjoyable: “*I do realise that I’m potentially doing myself some harm by continuing to play with all the risks factors that I have – so it’s got to be worth it, it’s got to be fun.” (YA004).* And whilst some participants conceded that their playing days may be numbered: “I’ve always said to myself that if it does happen again then that would be it for me – no more rugby” (YA011), others expressed a more fatalistic attitude towards their knee health: “Yeah my Grampy had arthritis in his knees, so I feel like I’m going to get it anyway” (YA005).

#### “It was a whole lifestyle overhaul for me”

For some participants, knee injury profoundly impacted their emotional wellbeing. In the short-term, participants described a loss of independence as they relied on others to support them with practical tasks (e.g., to drive them around), and frustration at the prospect of a lengthy period on the sidelines. Furthermore, time out of their sport, or the prospect of not returning to the sport, had affected their athletic identity and this had repercussions for their broader quality of life:

*“It certainly was very difficult, I suffered with a few mental health concerns, because it was that dedication, and that dedicating to sport that obviously being told you can’t do it anymore; it was a difficult change because it was a whole lifestyle overhaul for me.”* (YA008)

As well as the time and energy that participants had dedicated to the physical demands of their sport, they had also invested in it socially, forming close bonds to teammates or other people involved in that environment. This meant that their sense of personhood was intrinsically linked to their sport: “*that’s all I do really – play sport and have a beer”* (YA007). During the process of recovering from injury, some participants found solace in teammates, particularly those who had endured a similar injury experience: *“I do think yeah you need people around you to push you and that’s what’s been great with [name] because she’s sadly been through it – she’s been able to help me.”* (YA012). However, others found it difficult to reconnect to their sport following injury, with the association serving as a painful reminder of their absence:

*“So I went and watched my team play in the cup final at the end of the season. And that was the first time I had even been near a rugby ball since I had done it. I thought it would be fine. I didn’t even make it to half time, just mentally I couldn’t do it. So for now I have no intentions of going back, but maybe in the future, I’m not sure.”* (YA010)

The impact of sport cessation was felt hardest amongst those who had the most invested in their sport prior to injury: *“To go from being team captain where I was very involved to being out of the game has been difficult”* (YA011). And, for some, serious knee injury had meant having to re-evaluate their future, and any prospect they may have had of a sporting career: *“I was playing at British college levels at the time, and I was trying to work my way up to rugby as a career, but in terms of that, it wasn’t meant to be. That was the life I was given I suppose”* (YA008). For others, there was a sense that their playing time had been cut short, and that their peers carried on in their absence: “*it does get you down after a while, like I am only 31, everyone is still playing aren’t they?* (YA007).” Finally, there was an acceptance amongst participants that knee injury necessitated a change in their approach to sport and exercise. This meant reducing the frequency and intensity at which they played their sport or participating in different types of sport or exercise that were likely to attenuate the risk to knee health:

*“It has changed my exercise. For example, I would like to do long distance running, but I don’t trust that I would be able to do that without hurting my knee more; so I wouldn’t I don’t do that. I do 5k tops and stop”.* (YA002)

### Theme 3. Motivation to conserve knee health

#### “Do what you can, while you can”

There was a widely held belief amongst participants that PA is good for your health. In a general terms, PA was perceived as important for ensuring future joint health: *“If I keep training and keep myself nice and loose, stay active and then hopefully I can stay on my feet a lot longer than people do nowadays”,* whilst inactivity was considered to have a deleterious effect: “*a lot of people get these kind of injuries and then go in to a downward spiral and allow things like arthritis to take over”* (YA009). In addition, some participants recognised that a healthy body weight and increased strength in the muscles around the knee joint may reduce (or prevent) the occurrence of knee symptoms: *“I lost a lot of weight, I think that helped reduce it. And doing the lifting because the muscles are strong around the joint. I think that’s prevented it”* (YA003). There was also an implicit belief amongst some participants that by continuing to be physically active and keeping the muscles strong around the joint, they had some control over their knee health. As a result, they were prepared to recalibrate their exercise regimen if that meant they could safeguard their knee health:

*“When I first did it, I like to run, running’s my thing and I found that weights were always very hard to do. Whereas now, I will do every weight session and I’ll probably do an extra weight session knowing that that's good. Yeah, I just want to keep everything strong basically.”* (YA013)

Attitudes towards PA had also been shaped by older people and ex-players associated with sports clubs who warned about the problems with neglecting knee health: *“Being part of a rugby club, you see a lot of the ex-players coming up with walking sticks and saying that they let themselves get that way”* and advising that “*you gotta keep yourself moving, if you stop then things are going to seize up”* (YA009). Furthermore, there was a persistent view amongst participants that it is important to make the most of what you have, reinforced by the sage advice handed down by elders: “*there’s this old guy that comes into the gym, I chat to him all the time and he says ‘do what you can, while you can’. So, I do that within reason”* (YA010).

#### “I just like having that something that I can say is mine”

Participants were intrinsically motivated to recover from injury and maintain their knee health. This stemmed from a deep-rooted desire to stay physically active, an appetite for competition, and the sense of accomplishment that they associated with sport and exercise: “*I really like doing my sport, I enjoy the competitiveness of it, and that sense of achievement when you have really done well”* (YA003). The incentive of returning to previous performance levels was a powerful motivator: “*My main motivation has been getting back to playing again”* (YA011), and participants described how they set goals for themselves to facilitate their return to sport: “*I always set myself goals. It might be like ergo[meter] scores or times and stuff. I always know what I want to aim for and what I’ve done – and I know what I can do if I push hard”* (YA001). Some participants reported that they were motivated by the enjoyment of sport and exercise, and that this had implications for their wellbeing, including the capacity to shift the mind’s focus from day-to-day issues that may be troubling them – a form of escapism:

*“I think I just find it really enjoyable. I think it’s good for my mental wellbeing, having something that you are only focussing on that thing. I think it can often be when you are doing kind of sport or activity that it takes you away from other things, or being mindful I guess.”* (YA003)

Participation in sport and exercise was also perceived as confidence building, as well as a way in which anxieties could be addressed, giving some a sense of control over their life, a sense of ownership, and a way in which they could channel some of life’s challenges: *“It gives me something that is mine, it helps me with my anxieties and things … I just like having that something that I can say is mine as opposed to having something that I am doing for someone else”* (YA008). Broader connotations with health and wellbeing were expressed too, with sport and exercise perceived as a way of life: *“I think health is a huge factor, but I just enjoy it, I have been raised in an active family, and it’s all I know really, I couldn’t imagine sitting around doing nothing all day.”* (YA009).

Finally, participants articulated the importance of safeguarding their knee health in order that they could continue to stay physically active into the future, even if this meant that they had to adjust the type of exercise they did: *“If I can’t play football, I’d like to go and maybe do a bit of jogging, just to be able to use my legs as much as possible …. just simple things like that you start thinking about don’t you?”* (YA007).

### Theme 4. Opportunities for supplementary support

#### “It could be a diagnosis kind of thing”

Participants discussed how they could become better equipped to take control over their own knee health. This required access to resources that gave them the information necessary to monitor, manage, and treat symptoms where necessary, as well glean advice on conserving knee health to reduce the risk of future problems. Given the prominence of smartphone technology in daily activity, some participants envisaged a tool to track and manage knee symptoms: *“an app would be really useful in terms of knowing what’s normal and what’s not, thinking about flexion and that sort of thing, and swelling – how to deal with that*” (YA004). If necessary, it could then signpost users to an appropriate course of action (e.g., exercise mediation, exercise programmes to help stabilise the joint, referral to a healthcare professional). Tracking functionality would enable users to record progress and potentially identify the link between symptoms and their correlates *“because you do forget, you forget what symptoms you’re feeling in relation to what you did, and whether there’s any correlation*” (YA004). It was intimated that this could help to reassure users about symptoms or give users the confidence to manage their own exercise or PA programmes: *“If you are experiencing this, that, or the other, then that is just normal for someone who has had an injury, but if you are experiencing this or that, then it’s not quite so normal and may be you need to seek more help or something”* (YA006). In addition, it was suggested that an app could assess symptoms, including pain, using a standard questionnaire, and that this could help guide appropriate treatment: *If you were still getting trouble, like pains in your knee … it would have a set of questions, then it could be a diagnosis kind of thing*” (YA005).

As well as a tool to provide reassurance and guidance, participants posited an educational component to the smartphone app. This could help users to understand the nature of their knee injury: *“to make it as clear as possible, it would need to have a bit of everything, so videos, pictures, descriptions*” (YA010) and suggest exercises to strengthen the muscles around the knee, reduce the risk of re-injury and optimise knee health over the long-term:

*“I would like something where I could visually see how the exercise looks, with an explanation of what I should be feeling … like the steps to doing it and what muscles I am supposed to be engaging, along with repetitions and sets”.* (YA003)

Participants emphasised the importance of conveying the benefits of specific exercises and how adherence could support their long-term joint health: “*having a clear idea of what can I do to maintain the strength in the knee as best as I can into the future and what’s the benefit of that, specific and clear information”* (YA002). Furthermore, it was widely acknowledged that a self-management tool would need to be personalised, giving users the opportunity to tailor programmes to their individual needs: *“We’re all different and I’m going to have weaknesses in some areas where people are going to be quite strong, and vice versa, so being able to build your own programme within the app would be really handy”* (YA003).

#### “I wasn’t the only one having problems”

The vagaries of sport-related knee injury and the upheaval that it can cause in the various facets of an individual’s life prompted participants to seek solace from those who had gone through a similar experience. For those who participated in team sports, there were often others in the club who had experienced knee injury: “*I was very fortunate – there was another girl who had the same thing done. So we were the same, and I guess being around other people in the gym, they could tell you a bit more about what to do”* (YA012). However, others turned to social media for peer support: *“People post their stories of what happened and how they’re coping and what they’re doing. And they ask people questions and other people say what they found useful – what worked for them”* (YA011). Some participants articulated the sense of isolation that can follow knee injury and found comfort (and inspiration) in others’ experiences:

*“I did find a really useful group on Instagram called #ACLclub – it really helped me because it made me realise that I wasn’t the only one having problems or having the same frustrations. It made me feel less isolated and less on my own.”* (YA011)

As well helping individuals cope post-injury, social media was perceived as a source of inspiration for more creative exercise regimens: “*Instagram is quite good, I found some accounts which had exercises on, which were a bit more creative with things that you have to do”* (YA012). Social media also enabled access to a greater choice of exercise programmes that offered alternatives to standardised programmes available through traditional pathways: “*It’s given me ideas of how to make it sport-specific and a little less boring”* (YA003). Finally, participants discussed the benefits of motivational messaging, which they found particularly effective when they were struggling to stay self-motivated. For some, comparisons with injury-stricken peers helped to frame their own goals: “*I like going off statistics, so like if you do this plan for six weeks it’s proven that x-amount of people go back in x-amount of time, that definitely pushes me in the direction to do it”* (YA009).

## Discussion

The purpose of this study was to explore young adults’ experiences of sport-related knee injury and opportunities to support self-management of knee health. Four common themes were identified: [1] perceptions of current care provision; [2] long-term impact of knee injury; [3] motivation to conserve knee health; and [4] opportunities for supplementary support. Overall, participants described how the post-injury care they received was inconsistent and insufficient. Some participants reported receiving delayed diagnoses, as well as conflicting treatment advice, and standardised follow-up care that did not always meet the needs of young adults. This resulted in frustration and feelings of uncertainty amongst some participants, which continued long after the time of injury. Delays and inflexibility also prompted self-referral to private healthcare. There was broad consensus over the lack of long-term care provision, with issues such as chronic knee pain and stiffness persisting for months and years after standard treatment ceased. Participants were reluctant to report symptoms to their GP for fear of wasting time. Furthermore, knee injury had left participants conflicted about their participation in sport and exercise, fearing re-jury and wary of their knee health. In some cases, it also had wider implications for their emotional wellbeing, impacting on both their athletic and social identities. Despite this, there was a general acceptance of the benefits of PA in helping to safeguard joint health and an understanding of the risks associated with physical inactivity. Implicit motivations for sport and exercise participation were apparent: participants coveted a return to previous performance levels, they enjoyed competition, they found it to be confidence-building, and a form of escapism. Finally, participants discussed how a smartphone app could supplement face-to-face care through activity tracking, symptom monitoring, knowledge and advice provision, and peer support.

The expression *“Nobody says to you ‘come back in six months and we’ll see how you’re doing’”* captures the main findings of the current study within the context of the long-term impact of knee injury on young adults. When standard treatment ceased, participants were unsure of how to manage their knee health, whether or not they had knee-related symptoms and, in some cases, emotional wellbeing had been adversely affected. The limited care pathways currently available to patients in the UK do not adequately address their needs, with clear implications for longer-term prevention of PTOA or management of PTOA across the disease continuum. Post-injury, current evidence recommends that people be supported to [1] maintain a weight and [2] restore strength, balance, and healthy movement patterns that facilitate recommended levels of exercise, but a better understanding of the psychosocial factors involved (e.g., loss of athletic identity) is required [14]. Addressing individuals’ informational needs post-injury (including strategies that support physical and emotional wellbeing) has the potential to establish healthful behaviours that reduce the risk of PTOA or the severity of disease progression. In symptomatic populations, there are numerous international guidelines for OA management [13, 25], as well as a broad consensus on core therapies [13]. However, there is a huge disparity between current care and general OA treatment recommendations [26] – and therefore a clear need for interventions or programmes to bridge this gap.

The issue of knee confidence post-injury which emerged in the current study is consistent with two recently conducted qualitative studies, which found that respondents had significant ongoing concerns about returning to sport and an underlying fear of re-injury [12, 27]. This echoes a further study by Tjong at al. (2014), which found that fear was the most commonly reported reason for not returning to sport following ACL reconstruction [28]. Whilst this has potential repercussions for continuing engagement in PA, which a mitigating factor in the development of PTOA, fear itself may also prompt positive health behaviours. Regarding fear of re-injury following ACL reconstruction, Filbay et al. (2016) posit that this could serve as protective mechanism for optimising future knee health, though acknowledge that longitudinal studies would be needed to test this hypothesis [12]. In common with research conducted by Ezzat et al. (2018), participants in the current study expressed motivation and determination to meet the challenges that knee injury had initiated [27]. Their athletic and social identities prompted continued sport and exercise participation, whether for a return to competition or in the knowledge that it would benefit long-term knee health. Additionally, participants expressed the key support of teammates and Physiotherapists [27], as well as support from peers who had similar experiences.

The descriptions that participants gave of self-managing their knee health resonate with the concept of need-supportive versus need-frustrating environments as set out in Self-Determination Theory (SDT) [29]. According to SDT, individual behaviour can be regulated through either the satisfaction or frustration of three basic psychological needs of autonomy (feeling ownership of actions), relatedness (feeling connected to others) and competence (feeling capable to operate effectively). A need-supportive environment is one in which the individual perceives adequate support for making choices (autonomy support), for challenge and growth (competence support) and for being cared for and accepted (relatedness support) [30]. Moreover, needs support has been shown to predict autonomous forms of motivation (self-determined), including intrinsic regulation (e.g., I enjoy competing) and identified regulation (e.g., I exercise because it is good for my health) [29]. Individuals functioning in a more autonomous motivation manner report enhanced health and wellbeing, persistence, creativity, and better performance [29, 31]. SDT has been widely used to understand PA and exercise engagement and people’s persistence as documented by Teixeira et al. (2012), whose systematic review on the association between PA and exercise and SDT principles showed the relevance of autonomous motivation in nurturing PA [32]. Additionally, a meta-analysis of SDT related to a broad range of health contexts demonstrated that it is a viable conceptual framework in which to study antecedents and outcomes of motivation for health-related behaviours [33]. Such findings suggest that SDT could become a foundation for the development of self-management interventions within health promotion and healthcare contexts, including those which promote PA [29, 34]. Thus, efforts by healthcare professionals (GPs, Orthopaedic Consultants, Physiotherapists) to promote joint health, wellbeing and quality of life might benefit from including principles of SDT in delivering their messages.

This study canvassed the views of young adults because they represent the most underserved demographic with respect to OA, and for whom there is the greatest potential for maximising quality of life across the lifespan. Participants were recruited on the basis that they identified as sports participants and had experienced a sport-related knee injury. A limitation of the study was the possibility of recall bias, given the length of time since knee injury. Moreover, as with all qualitative research, our participant sample is unlikely to be representative of all people who have experienced knee injury. For instance, although data saturation was reached within the recruited cohort, knee injury pathology and associated symptoms are heterogenous by their very nature.

### Implications for future research

The current study was designed to capture the full spectrum of personal experiences following acute knee injury, providing insights into changes in knee-related quality of life over time that have not previously been accounted for in traditional quantitative methods of research. It highlights the need to improve access to appropriate care for young adults post-knee injury to address the perceived paucity in care provision and the potential for long-term impact on quality of life. This could be achieved by providing symptom self-management tools and a decision tree to guide their care-seeking behaviours, including signposting them to appropriate care at the appropriate time to minimise the impact of the disease on their health and related quality of life. Further research should promote collaboration between clinicians and researchers in determining appropriate treatment pathways, fostering high quality education and service provision through the development of an evidence-informed, interactive platform specifically created to meet the end-users’ needs. In parallel, there is also a need for clear evidence-based guidance on the best strategies for reducing the risk of PTOA or the severity of its symptoms, as well as interventions that can effectively deliver treatment and / or behaviour change recommendations that are tailored to individuals.

## Conclusion

Sport-related knee injury can have a profound and lasting impact on individuals’ health and wellbeing. Participants reported that current care provision is limited and does not account for the management ongoing knee symptoms or the impact on their quality of life, with clear implications for the delay or prevention of PTOA. This research highlights the areas in which particular attention might be focussed regarding self-management of knee health: activity tracking and symptom management, information and advice dissemination, and peer support.

## Supplementary information

**Additional file 1. **INTERVIEW TOPIC GUIDE: *“Exploring young adults’ experiences of managing knee health following a sport-related knee injury”* [Version 1.1].

## Data Availability

No additional data are available. All data related to this study are included in this submission, either in tables in the manuscript or in supplementary files.

## Abbreviations

ACL: Anterior cruciate ligament
GP: General practitioner
MCL: Medial Collateral Ligament
MRI: Magnetic resonance imaging
NHS: National Health Service
OA: Osteoarthritis
PA: Physical activity
PCL: Posterior cruciate ligament
PTOA: Post-traumatic osteoarthritis
SDT: Self-determination theory

## Footnotes

1 The NHS is a publicly-funded national healthcare system for England, and one of the four National Health Services operating across the UK’s constituent countries. Healthcare is provided to all legal residents, with most services free at the point of access.
