# Supplementary material for: Nobody says to you “come back in six months and we’ll see how you’re doing”: a qualitative interview study exploring young adults’ experiences of sport-related knee injury
Source: BMC Musculoskelet Disord. 2020 Jul 1;21:419. doi: 10.1186/s12891-020-03428-6 (PMC7329431; doi:10.1186/s12891-020-03428-6)
Supplement: Supplementary file 1 — Additional file 1. INTERVIEW TOPIC GUIDE: “Exploring young adults’ experiences of managing knee health following a sport-related knee injury” [Version 1.1]. [file 12891_2020_3428_MOESM1_ESM.docx]

**Appendix 1**

**INTERVIEW TOPIC GUIDE: *“Exploring young adults’ experiences of managing knee health following a sport-related knee injury”* [Version 1.1]**

| 1. **Background**   “Could you tell me about your knee injury and how you initially dealt with it?” | **Explore:**   - Injury experience - Rehabilitation and journey to recovery - The process of returning to sports - Any injury-related symptoms that are still experienced (knee pain, stiffness) |
| --- | --- |
| 1. **Techniques / strategies used**   “Can you tell me about the support you have been given by healthcare professionals (e.g., physiotherapists, doctors)? | **Explore:**   - Professional support / advice sought - Advice and guidance given - Satisfaction with care and support provided following the injury and through recovery - Any ideas around additional support that could have been given to aid rehab and ongoing knee health |
| 1. **Role of self-management in rehabilitation and ongoing conservation of joint health**   “What have you done to help improve your knee health following your injury?” | **Explore**   - Attitudes and beliefs about physical activity following the injury - Motivation - Psychological approaches - Self-management strategies used - Examples of strategies which have / haven’t worked - Any protective measures taken to safeguard knee health |
| 1. **Outcomes**   “What impact has your knee injury had on your life in general?” | **Explore**   - Impact on quality of life - Physical activity choices and preferences following injury - Perception of future knee health |
